# Population pharmacokinetics and pharmacogenomics of edoxaban in Japanese adults with atrial fibrillation

**DOI:** 10.1186/s40780-025-00453-2

**Published:** 2025-06-02

**Authors:** Satoshi Ueshima, Daiki Hira, Sayana Matsuda, Rio Michihata, Yohei Tabuchi, Tomoya Ozawa, Hideki Itoh, Moritake Iguchi, Masaharu Akao, Takanori Aizawa, Asami Kashiwa, Satoshi Shizuta, Takeru Makiyama, Yoshihisa Nakagawa, Minoru Horie, Tomohiro Terada, Toshiya Katsura

**Affiliations:** 1 College of Pharmaceutical Sciences, Ritsumeikan University, 1-1-1 Noji- higashi, Kusatsu, Shiga 525-8577 Japan; 2 Department of Pharmacy, Shiga University of Medical Science Hospital, Seta Tsukinowa-Cho, Otsu, Shiga 520-2192 Japan; 3 Department of Cardiovascular Medicine, Shiga University of Medical Science, Seta Tsukinowa-Cho, Otsu, Shiga 520-2192 Japan; 4 Department of Cardiology, National Hospital Organization Kyoto Medical Center, 1-1, Mukaihata-cho, Fukakusa, Fushimi-ku, Kyoto, 612-8555 Japan; 5 Department of Cardiovascular Medicine, Kyoto University Graduate School of Medicine, 54 Shogoin Kawahara-Cho, Sakyo-Ku, Kyoto, 606-8507 Japan; 6 Present Address: Department of Clinical Pharmacology and Therapeutics, Kyoto University Hospital, 54 Shogoin Kawahara-cho, Sakyo-ku, Kyoto, 606- 8507 Japan; 7 Present Address: Division of Patient Safety, Hiroshima University Hospital, 1-2-3 Kasumi, Minami-ku, Hiroshima, 734-8551 Japan; 8 Present Address: Department of Cardiology, Niigata City General Hospital, 463-7 Shumoku, Chuo-ku, Niigata, 950-1197 Japan

**Keywords:** Edoxaban, Pharmacokinetics, Pharmacogenomics, Non-linear mixed-effects modeling, Atrial fibrillation

## Abstract

**Background:**

Edoxaban is used as an anti-coagulant to prevent cardioembolic infarction, deep vein thrombosis, and pulmonary embolism. Edoxaban pharmacokinetics have been reported to be affected by several factors such as renal function, age, body weight, and the concomitant use of P-glycoprotein inhibitors. However, the relationship between genetic polymorphisms in drug metabolizing enzymes and transporters and the inter-individual variability of edoxaban pharmacokinetics in patients with atrial fibrillation (AF) remains unclear. Additionally, there is little information concerning PPK analysis using real world data. In this study a population pharmacokinetic and pharmacogenomic analysis was conducted to clarify covariate factors affecting the edoxaban pharmacokinetics in Japanese adult AF patients.

**Methods:**

One hundred and thirty-one blood samples were collected from 131 patients. The edoxaban pharmacokinetic profile was described by a one-compartment model, and pharmacogenomic data were stratified according to *CYP3A5* (CYP3A5*3) and *ABCB1* (*ABCB1* 1236 C > T, 2677G > T/A, and 3435 C > T) polymorphisms. A non-linear mixed-effects modeling software (NONMEM™) was used to evaluate the effects of patient characteristics and genetic polymorphisms on the edoxaban pharmacokinetics.

**Results:**

The apparent oral clearance (CL/F) of edoxaban was estimated, and the apparent volume of distribution was fixed at the reported value. The CL/F of edoxaban was correlated non-linearly with creatinine clearance (CLcr), wherein the population mean CL/F for a typical patient (CLcr = 61.8 mL/min) was estimated to be 28.2 L/h. Other clinical laboratory data and genetic polymorphisms, excluding CLcr, did not affect the edoxaban pharmacokinetics.

**Conclusions:**

These results suggest that genetic polymorphisms of *CYP3A5* and *ABCB1* are not considered intrinsic factors affecting edoxaban pharmacokinetics in Japanese adult AF patients. Similarly to previous studies, renal function affects its pharmacokinetics. These findings may provide useful information for individualized anticoagulant therapy with edoxaban to prevent adverse events without reference to genetic polymorphisms of *CYP3A5* and *ABCB1*.

## Background

Atrial fibrillation (AF) is a common arrhythmia known to be associated with the major risk factors for cardioembolic infarction [1]. The Hisayama Study, a prospective cohort study in Japan, demonstrated that the 5-year survival rate for cardioembolic infarction was lower than for lacunar or atherothrombotic infarction [2]. Edoxaban directly inhibits the activated coagulation factor X (FXa), and has been used as an anti-coagulant for cardioembolic infarction, deep vein thrombosis, and pulmonary embolism [3–5]. The ENGAGE AF-TIMI 48 clinical trial demonstrated that in AF patients, edoxaban had comparable efficacy and safety to warfarin, which has long been used as an anti-coagulant for vitamin K [4]. Unlike warfarin, it is difficult to adjust the edoxaban dosage according to anti-coagulant activity indices — such as activated partial thromboplastin time, prothrombin time, international normalized ratio of prothrombin time, and intrinsic FXa activity — as therapeutic windows of edoxaban regarding anti-coagulant activity indices have not been established [6, 7]. Several exposure-response studies suggest that the plasma trough concentration of edoxaban is associated with the incidence of bleeding, systemic embolic events, and ischemic stroke [8–10]. Therefore, monitoring the plasma trough edoxaban concentration (rather than the anti-coagulant activity indices) in AF patients may be clinically useful to ensure safer and more effective therapeutic dosages. Additionally, it is clinically significant to clarify the pharmacokinetic properties of edoxaban.

Edoxaban is a known substrate of P-glycoprotein (P-gp; ABCB1; expressed in the liver, kidneys, and small intestine) [7, 11] and the cytochrome P450 (CYP) isozymes 3A4/5, which are primarily expressed in the small intestine and liver [7, 11, 12]. Therefore, the inter-individual variability of edoxaban pharmacokinetics may be affected by the expression and/or function of these proteins. Pharmacokinetic and pharmacogenomic studies of FXa inhibitors have focused on the effects of single nucleotide polymorphisms in genes encoding drug transporters and metabolizing enzymes [13–16]. For edoxaban, *ABCB1* polymorphisms were reported to have no effect on the edoxaban pharmacokinetics, while organic anion transporting polypeptide 1B1*15 polymorphism, a hepatic uptake transporter, was reported to affect the plasma concentration of M-4, an edoxaban metabolite [17, 18]. Nevertheless, the relationship between the variability of edoxaban pharmacokinetics and genetic polymorphisms of drug transporters and drug-metabolizing enzymes remains unclear.

Population pharmacokinetic (PPK) analysis is a quantitative method for explaining the inter-individual variability in drug concentrations, in which population pharmacokinetic parameters of drugs enable us to predict its concentrations using the Bayesian estimation for precision medicine. Several PPK studies of edoxaban using clinical trial data have shown that the variability of edoxaban pharmacokinetics is due to renal function, age, body weight, and the concomitant use of P-gp inhibitors (such as quinidine, verapamil, dronedarone, ketoconazole, and erythromycin) [8, 19–22]. However, a PPK and pharmacogenomic analysis of edoxaban has not been reported in AF patients and there is little information concerning PPK analysis using real world data.

This study aimed to identify covariate factors, such as the *ABCB1* and *CYP3A5* polymorphisms as well as clinical laboratory data, affecting edoxaban pharmacokinetics using a PPK analysis.

## Methods

### Patients and study design

Adult Japanese inpatients and outpatients with AF who were treated with edoxaban at Shiga University of Medical Science Hospital, National Hospital Organization Kyoto Medical Center, and Kyoto University Hospital between January 2017 and August 2019 were enrolled in this study. Edoxaban tablets (Lixiana^®^; Daiichi Sankyo Co. Ltd, Tokyo, Japan) were orally administered to all patients once daily at a dosage of 15–60 mg/day. For a total of 131 patients, blood samples were collected in 3.2% citrated tubes at a single point (9–47 h after the last edoxaban dose at steady state). Clinical data, including age, body weight, sex, serum creatinine (Scr), creatinine clearance (CLcr), alanine aminotransferase (ALT), and aspartate aminotransferase (AST) levels, and the concomitant use of CYP3A4 and/or P-glycoprotein inhibitors or inducers, were collected retrospectively from patients’ electronic medical records. The Cockcroft-Gault equation was used to calculate CLcr [23]. Physicians and pharmacists obtained data regarding drug compliance during each hospital visit or hospitalization to confirm drug exposure; patients who exhibited poor drug compliance according to their assessment, or who had no record of these measurements, were excluded from the analysis.

### Edoxaban assay

Plasma specimens from patients were separated via centrifugation (2,500 g for 15 min at 4 °C) and stored at − 80 °C until analysis. The plasma concentrations of edoxaban were measured with high-performance liquid chromatography with electrospray ionization-tandem mass spectrometry by a validated external commission company (Shin Nippon Biochemical Laboratories Ltd. Pharmacokinetics and Bioanalysis Center, Wakayama, Japan). The lower limit of quantification for edoxaban was 1 ng/mL, and the inter-day variation of the analysis was < 4.4%.

### Genotyping

Genomic DNA was extracted from blood samples using the DNA Extract All Reagents Kit (Applied Biosystems, Waltham, MA, USA). The CYP3A5*3 (rs776746), and *ABCB1* 1236 C > T (rs1128503), 2677G > T/A (rs2032582), and 3435 C > T (rs1045642) polymorphisms were determined using a real-time polymerase chain reaction system (StepOnePlus™; Applied Biosystems), as previously described [13].

### PPK modeling

PPK modeling was performed using the non-linear mixed-effects modeling (NONMEM) software (version 7.3.0; ICON Public Limited Company, Dublin, Ireland) via the first-order conditional estimation with interaction method. As there were no data on the absorption phase, a 1-compartment model without first-order absorption was employed for pharmacokinetic modeling to describe the plasma concentration-time profiles of edoxaban after oral administration (ADVAN1 TRANS2). Using an exponential error model, considering the residual variability (*ε*), the relationship between observed and predicted edoxaban concentrations was described as follows:

1 $${C_{ij}}=\overline {{{C_{ij}}}} \cdot \exp \left( {{\varepsilon _{ij}}} \right)$$

C_ij_ and $\overline {{{C_{ij}}}}$ are the observed and predicted edoxaban concentrations in the *j*th record of the *i*th patient, respectively. The *ε*_ij_ was considered as random effects with a mean of 0 and variances of *σ*^2^. The *ε*_ij_ in the error model was expressed as the percent coefficient of variation (CV%). The coefficient of variation of *ε*_ij_ was fixed at the previously reported value of 58.7% by considering that for healthy subjects (14.6%) and the increment for AF patients (44.1%) [22]. The apparent oral clearance (CL/F) of edoxaban was estimated as the pharmacokinetic parameter, which was then described using the population mean parameters (*θ*), as in the following equation:

2 $${\left( {CL{\text{/}}F} \right)_i}={\theta _1} \cdot \exp \left( {{\eta _{1i}}} \right)$$

(CL/F)_i_ is a CL/F of the ith patient, and *η*_1_ is the inter-individual variability of CL/F. Similarly to *ε*_ij_, the *η*_1_ was also considered as random effects with a mean of 0 and variances of *ω*^2^. The *η*_1_ in the error model was expressed as the percent coefficient of variation. Conversely, the apparent volume of distribution (Vd/F) was described by following the allometric equation using the previously reported value [22] with the exponent fixed to 1 due to a lack of individual data:

3 $${\left( {Vd{\text{/}}F} \right)_i}={\theta _2} \cdot \left( {\frac{{B{W_i}}}{{70}}} \right)=336.4 \cdot \left( {\frac{{B{W_i}}}{{70}}} \right)$$

BW_i_ is the body weight of the *i*th patient. The *θ*_2_ was fixed at the 336.4 L by considering the typical value of the central volume of distribution (193 L) and the fold-change for Asians (1.743 fold) [22]. Hereafter, the basic model for subsequent covariate analysis was constructed by the combination of these equations.

The influence of CLcr was first evaluated in the covariate model to develop a PPK model of edoxaban, as renal function is reported to be related to the CL/F of edoxaban [8, 19–22]. Edoxaban is known to be eliminated in the kidney and metabolized in the intestine and liver [11], and thereby the CL/F of edoxaban was evaluated as the sum of the apparent renal (CL_R_/F) and apparent non-renal (CL_NR_/F) clearances, as follows [20]:

4 $${\left( {CL{\text{/}}F} \right)_i}={\left( {C{L_R}{\text{/}}F} \right)_i}+{\left( {C{L_{NR}}{\text{/}}F} \right)_i}$$

A covariate analysis was performed to evaluate the effect of CLcr on CL_R_/F using the following equations:

5 $${\left( {C{L_R}{\text{/}}F} \right)_i}={\theta _1} \cdot {\left( {\frac{{CLc{r_i}}}{{CLc{r_{median}}}}} \right)^{{\theta _3}}}$$

6 $${\left( {C{L_R}{\text{/}}F} \right)_i}={\theta _1} \cdot \left( {\frac{{CLc{r_i}}}{{CLc{r_{median}}}}} \right)$$

CLcr_i_ and CLcr_median_ are the CLcr of the ith patient and median CLcr, respectively, while *θ*_3_ is the population mean estimate. Based on the lowest objective function value (OBJ), calculated using the NONMEM software, the covariate models incorporating the CLcr value were selected. After performing the covariate analysis for CLcr, continuous covariates — such as ALT and AST levels, age, and body weight of patients for the parameter CL_R_/F and CL_NR_/F — were normalized by each population median using the following power function or linear model:

7 $${P_i}={\theta _4} \cdot {\left( {\frac{{CO{V_i}}}{{CO{V_{median}}}}} \right)^{{\theta _5}}}$$

8 $${P_i}={\theta _4} \cdot \left( {\frac{{CO{V_i}}}{{CO{V_{median}}}}} \right)$$

P_i_ is CL_R_/F or CL_NR_/F, and COV_i_ and COV_median_ are the covariate of the ith patient and median of the covariate, respectively; *θ*_4_ and *θ*_5_ are population mean estimates. For categorical covariates, such as sex, concomitant CYP3A4 and/or P-glycoprotein inhibitor use, genetic polymorphisms in ABCB1 and CYP3A5, and haplotype of ABCB1 with 2677G > T/A and 3435 C > T, the dichotomous parameter A is set to 0 for the normal classification, and 1 for the other classification in each individual, as follows:

9 $${P_i}={\theta _4} \cdot \theta _{6}^{A}$$

For example, in the case of genetic polymorphisms, if a patient had a specific genotype, the parameter A was set to 1; if not, it was set to 0. Covariates were added to the basic model or the model involving CLcr via a forward stepwise selection method; statistical significance was set at 0.05, with 1 degree of freedom if the OBJ decreased by > 3.84. Subsequently, using a backward stepwise selection method, covariates were removed from the full model; statistical significance was set at 0.01, with 1 degree of freedom if the OBJ decreased by > 6.63. In addition to decrease in OBJ values, covariates for the CL_R_/F and CL_NR_/F of edoxaban were also selected based on their 95% confidence intervals for the effect sizes (*θ*_3_ in Eq. 5, *θ*_5_ in Eq. 7, and *θ*_6_ in Eq. 9) in covariate models. In a forward or backward stepwise selection process, we judged that these covariate models did not affect these parameters of edoxaban if the 95% confidence intervals for *θ*_3_ and *θ*_5_, and *θ*_6_ included 0, 0, and 1, respectively.

### Model evaluation

To evaluate the adequacy of the models, the following goodness-of-fit plots were used: observed (OBS) versus population predicted value (PRED); OBS versus individual predicted value (IPRED); conditional weighted residuals (CWRES) versus time after the last dose; and CWRES versus PRED. To evaluate the final model for edoxaban, the prediction-corrected visual predictive check (pcVPC) and non-parametric bootstrap analysis were used. In the VPC analysis, 1,000 hypothetical datasets were simulated via random sampling using the NONMEM program. The median value and 90% prediction interval of the simulated concentrations were plotted using the observed concentrations. In the bootstrap analysis, 1,000 replication datasets were generated by random sampling using Perl-speaks-NONMEM version 4.7.0 [24]. The median and 95% prediction intervals using bootstrap analysis were compared, with the estimates of each PPK parameter obtained using the final model.

### Model-based simulation

The Monte Carlo simulation was used to evaluate the impact of CLcr on the steady-state trough concentration (24 h after the last dose) of edoxaban. Using the NONMEM program, 1,000 pharmacokinetic profiles were simulated for patients with various genetic polymorphisms and CLcr values (30, 60, 90, and 120 mL/min). The oral dosing schedules of edoxaban were set according to the package insert. The oral dosage of edoxaban was set to 30 mg/day if a patient had a CLcr value of 30 mL/min; otherwise, the dosage was set to 60 mg/day.

### Statistical analysis

Unless otherwise indicated, data were expressed as median values. The statistical significance of differences among 4 groups was analyzed using the Kruskal-Wallis test, followed by Dunn’s post hoc test using the Prism 6 software (GraphPad Software, San Diego, California, United States). The χ2 test was employed to evaluate the distribution of the polymorphic alleles based on the Hardy-Weinberg equilibrium. A probability value < 0.05 was considered statistically significant.

## Results

### Patient characteristics

In total, 131 observations from 131 patients were included in this study; their characteristics and the distribution of each genotype are summarized in Table 1. All allele frequencies in this study were consistent with the Hardy-Weinberg equilibrium.

**Table 1:** Clinical characteristics and demographics of AF Patients^a^

Number of patients	131
Number of measurements	131
Sex (male/female)	83/48
Age (years)	72.2 (35.2 − 92.6)
Body weight (kg)	61.4 (37.3 − 97.3)
Number of patients administered edoxaban (15 mg/day, 30 mg/day, 60 mg/day)	3, 70, 58
Plasma concentration of edoxaban (ng/mL)	19.5 (1.7 − 152.0)
Serum creatinine (mg/dL)	0.89 (0.56 − 2.00)
Creatinine clearance (mL/min)	61.8 (20.0 − 135.5)
Aspartate amino transferase (IU/L)	25 (11 − 92)
Alanine amino transferase (IU/L)	19 (5 − 77)
Number of patients treated with CYP3A4 and/or P-glycoprotein inhibitor	
Amiodarone, Diltiazem, Verapamil	5, 6, 5
Number of patients with *CYP3A5* and/or *ABCB1* genotypes	
*ABCB1* 1236 C > T (C/C, C/T, T/T)	56, 56, 19
*ABCB1* 2677G > T/A (G/G, G/T, G/A, A/A, T/A, T/T)	18, 20, 48, 23, 19, 3
*ABCB1* 3435 C > T (C/C, C/T, T/T)	43, 65, 23
CYP3A5*3 (*1/*1, *1/*3, *3/*3)	2, 38, 91

^a^ Data are presented as the number or median with the range in parentheses

### PPK modeling

The relationship between the CLcr and CL/F of edoxaban was first analyzed using the covariate model. When compared with the basic model, the OBJ values, with covariate models incorporating CLcr with Eqs. 5 and 6, significantly decreased by 89.8 and 69.6 respectively. Thus, the covariate model incorporating CLcr with Eq. 4 was the most reasonable to characterize the relationship between CL_R_/F and CLcr. Additionally, the stepwise forward inclusion and backward elimination method showed that CLcr significantly affected the CL_NR_/F of edoxaban, whereas covariates such as AST, ALT, concomitant CYP3A4 and/or P-glycoprotein inhibitors, *CYP3A5* and *ABCB1* genotypes, and haplotype of ABCB1 with 2677G > T/A and 3435 C > T did not significantly affect that of edoxaban. Additionally, there were no covariates affecting the CL_NR_/F of edoxaban in Eqs. 7 and 8.

Table 2 shows the final PPK parameter estimates of edoxaban in Japanese AF patients. The CL/F of edoxaban was estimated using the following PPK model:

**Table 2:** PPK parameter estimates for Edoxaban in AF patients^a^

Parameters	Original data		1,000 Bootstrap sample data	
	Mean	95% Confidence interval	Median	2.5th to 97.5th percentiles
$$CL{\text{/}}F\,\,(L{\text{/}}h)={\theta _1} \cdot {\left( {\frac{{CLcr}}{{61.8}}} \right)^{{\theta _3}}}$$				
${\theta _1}\,\,(L{\text{/}}h)$	28.2	26.9−29.5	28.1	26.6−29.3
${\theta _3}$	0.692	0.582−0.801	0.693	0.578−0.804
$$Vd/F\,\,(L)={\theta _2} \cdot \left( {\frac{{BW}}{{70}}} \right)$$				
${\theta _2}\,\,\left( L \right)$	336.4 fixed	−	336.4 fixed	−
Inter- and residual variabilities ^b, c^				
*ω*_1_ (CV%)	26.4 (27.4)	18.8−34.0	25.9	16.4−32.7
*σ* (CV%)	58.7 fixed	−	58.7 fixed	−

^a^The *θ*_2_ and *σ* values were set to the literature values [22]

^b^The *ω*_1_ and *σ* values were expressed as the coefficient of variations of the inter-individual variability for CL/F and residual variability, respectively

^c^This value was presented as the mean with the shrinkage (%) in parenthesis

BW; body weight; CLcr, creatinine clearance; CL/F, apparent oral clearance; Vd/F, the apparent volume of distribution; *θ*, population mean parameters

10 $$CL{\text{/}}F\,\,\left( {L{\text{/}}\text{h}} \right)=28.2 \cdot {\left( {\frac{{CLcr}}{{61.8}}} \right)^{0.692}}$$

The population mean of CL_NR_/F was constant, and calculated to be 0.01 L/h. Therefore, this value was judged to be negligible. A non-linear correlation existed between the CL/F of edoxaban and CLcr. The population mean of CL/F for a typical patient (CLcr value of 61.8 mL/min) was estimated to be 28.2 L/h, and the coefficient of variation of the inter-individual variability in CL/F was 26.4%.

### Model evaluation

**Fig. 1:**
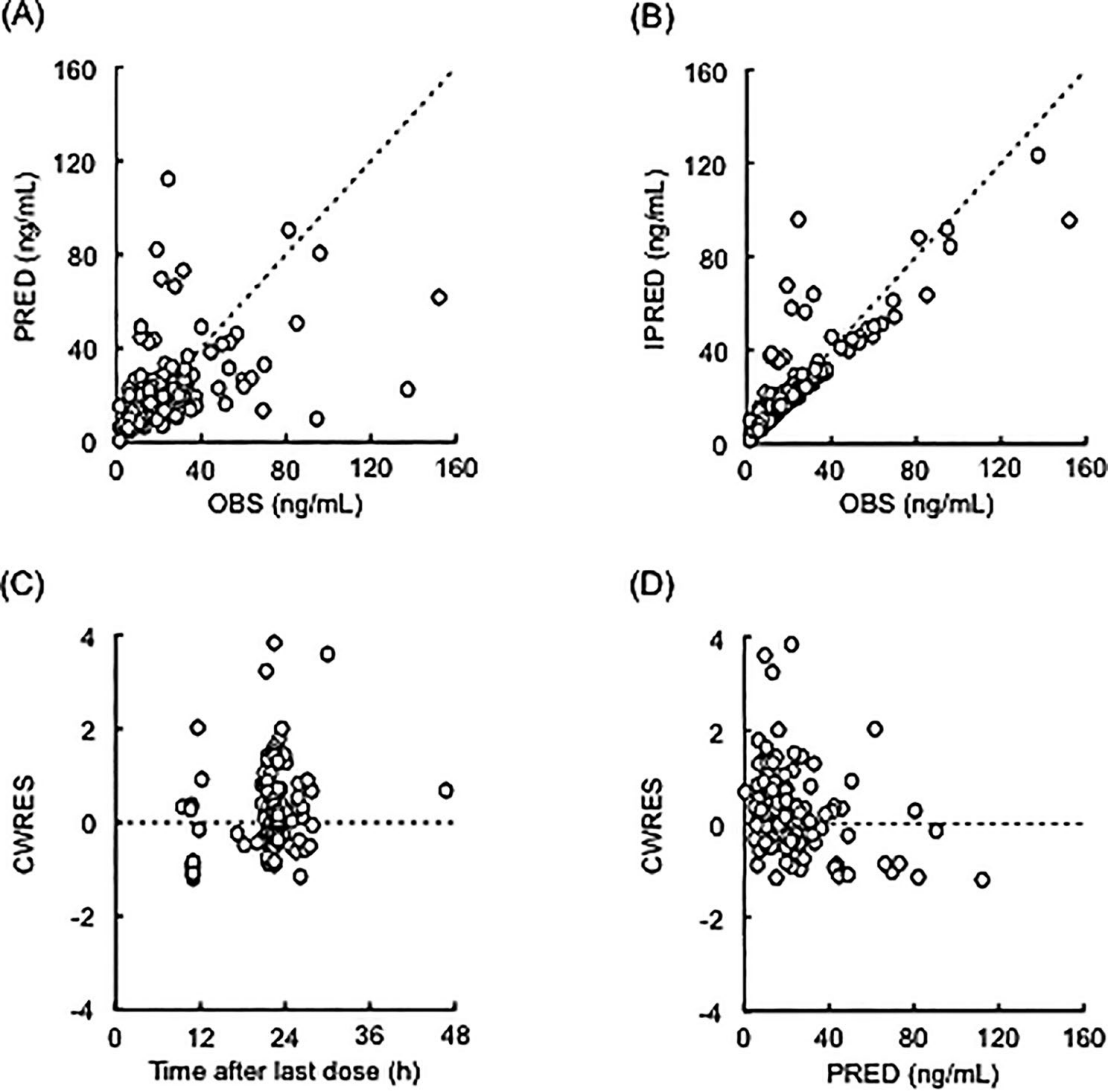
Goodness-of-fit plots for the final pharmacokinetic model of edoxaban. The observed plasma concentrations (OBS) versus population predictions (PRED; panel **A**) or individual predictions (IPRED; panel **B**) are shown, as well as conditional weighted residuals (CWRES) versus the time after the last dose (panel **C**) or PRED (panel **D**). Open circles show the observed values, and each dotted line shows a line of identity

**Fig. 2:**
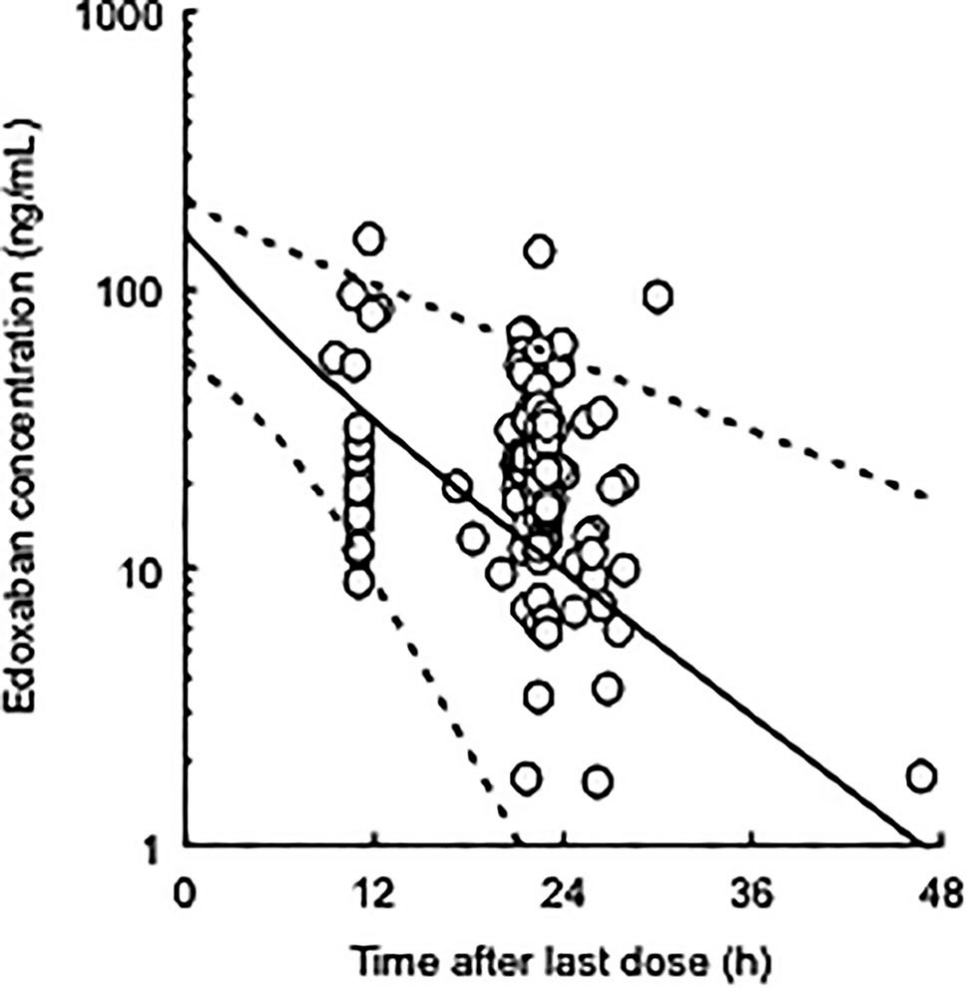
Prediction-corrected visual predictive check plot for the final pharmacokinetic model of edoxaban. Open circles show the observed concentrations. The top dotted, middle solid, and bottom dotted lines represent the 95th, 50th, and 5th percentiles, respectively, calculated from 1,000 simulated datasets

Figure 1 shows the goodness-of-fit plots for the final pharmacokinetic model. The OBS correlated with PRED, and the relationship between OBS and IPRED approached the line of identity. No obvious deviation was observed in the plots of CWRES against time after the last dose, and PRED. Additionally, the median values of the PPK parameters from 1,000 bootstrap re-samplings were similar to the mean estimates in the final pharmacokinetic model (Table 2). The pcVPC plot for plasma edoxaban concentrations versus time after the last dose indicated reasonable predictability of the final pharmacokinetic model (Fig. 2).

### Model-based simulation

**Fig. 3:**
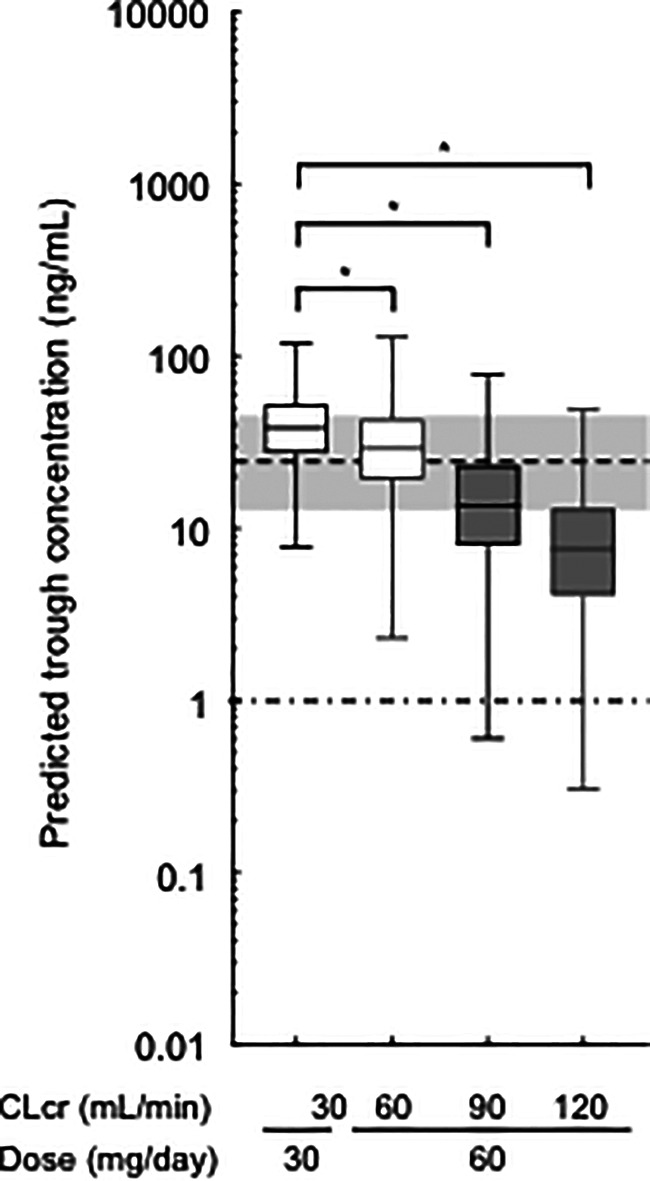
Simulations of trough concentrations of edoxaban using the final pharmacokinetic model. The 1000 replication datasets of a patient administering a typical dose of 30 or 60 mg once daily were simulated. These simulations were conducted using the final pharmacokinetic model. Box-and-whisker plots are presented according to three quartiles and the minimum and maximum values. The gray shaded area and the dotted line show the interquartile range and median trough concentration in Asians reported by Chao et al. [10], respectively. The chain line shows the lower limit of quantification (1 ng/mL) in this study. * P < 0.001 via the Kruskal-Wallis test, followed by Dunn’s multiple comparison test

Figure 3 shows the simulated steady-state trough concentrations of edoxaban using the final PPK parameters. These predicted concentrations were consistent with previously observed concentrations (median: 25.0 ng/mL, inter-quartile range: 13.9–47.4 ng/mL) [10].

## Discussion

In this study, we examined the impact of *ABCB1* and *CYP3A5* polymorphisms and clinical laboratory data on pharmacokinetic parameters of edoxaban via non-linear mixed-effects modeling. To the best of our knowledge, this is the first PPK and pharmacogenomic analysis of edoxaban to see if genetic polymorphisms of drug metabolizing enzymes and drug transporters affect edoxaban pharmacokinetics.

Multiple pharmacokinetic parameters could not be mathematically estimated due to only a single sampling data on the elimination phase. Previous studies have suggested that sampling points at peak concentration of drug and on the elimination phase were required to accurately estimate the means of Vd/F and CL/F, respectively, for a one-compartment model without first-order absorption [25–27]. Therefore, in this study, the CL/F of edoxaban was estimated whereas the Vd/F was fixed at the reported value for Asian in the clinical trial [22]. Several PPK studies of edoxaban have reported that the correlation between the CLcr and CL/F of edoxaban can be described using a linear [8, 20, 22] or power [19] model. As shown in Table 2, our analysis demonstrated a non-linear correlation between CL/F and CLcr, suggesting that our model is similar to those in previous studies.

The CL/F value of edoxaban for a typical patient (CLcr = 61.8 mL/min) was 28.2 L/h in this study, which is comparable to the population mean in previous PPK studies (24.7–32.1 L/h) [8, 19, 21]. The population mean of CL_NR_/F in this study was too small compared with that of CL_R_/F, in which the CL/F of edoxaban was approximated as its CL_R_/F. On the contrary, the reported CL_R_ value of edoxaban was estimated to be 10.7 L/h in healthy subjects after intravenous administration; this corresponds with 49.1% of its CL [28], suggesting that the CL_NR_/F of edoxaban is unable to be negligible. In this study, it was difficult to separately estimate the CL_R_/F and CL_NR_/F of edoxaban due to no collection of its urine and metabolite samples. The CL/F values of edoxaban in patients with normal renal function (CLcr = 100 mL/min), mild (CLcr = 60 mL/min) and severe (CLcr = 30 mL/min) renal impairment using our final pharmacokinetic model were estimated to be 39.3 L/h, 27.6 L/h, and 17.1 L/h, respectively. These values are comparable to previously reported values (normal renal function: 34.6 L/h; mild renal impairment: 24.8 L/h; severe renal impairment: 18.5 L/h) [22]. Additionally, bootstrap, pcVPC showed that the final pharmacokinetic model provided a robust and unbiased fit to the data (Fig. 2; Table 2). As shown in Fig. 3, simulation analysis suggested that the predicted trough concentrations of edoxaban in hypothetical patients with CLcr value of 90 and 120 mL/min using the final PPK parameters tended to be lower than the median of previously observed concentrations [10]. However, the predicted concentrations were considered to be generally consistent with previously observed concentrations.

In this study, AST and ALT levels did not affect the edoxaban pharmacokinetics, which is comparable with a clinical study on the effect of mild or moderate hepatic impairment on edoxaban pharmacokinetics [29]. These results suggest that the edoxaban dose for patients with mild or moderate hepatic impairment is similar to that for patients with normal hepatic function.

Considering an edoxaban CL_R_ value of 10.7 L/h in healthy subjects, and its protein binding value of 0.55 [22], the CL_R_ value of edoxaban corrected by the unbound edoxaban concentration was calculated to be 396. 3 mL/min, which is higher than the normal glomerular filtration rate of approximately 100 mL/min. Drug-drug interactions between edoxaban and P-gp inhibitors (quinidine, verapamil, amiodarone, dronedarone, cyclosporine, ketoconazole, and erythromycin) [30, 31] or inducers (rifampicin) [32] were observed in healthy subjects. These results suggest that the variation of the expression and/or function of ABCB1 can partly account for the variability of edoxaban pharmacokinetics. The effects of the *ABCB1* polymorphisms on pharmacokinetics of drugs remains controversial, even when the same drugs — such as cyclosporine [33–35], digoxin [36–38] and tacrolimus [39–41] — are evaluated. The effect of the haplotype with *ABCB1* 2677G > T/A and 3435 C > T polymorphisms on pharmacokinetics of drugs has been examined since these polymorphisms were known to show strong linkage disequilibrium [42]. However, contradictory results have been reported concerning the association of this haplotype with the pharmacokinetics of drugs such as cyclosporin [43, 44] and tacrolimus [39, 45, 46]. In the present study, the *ABCB1* 1236 C > T, 2677G > T/A, and 3435 C > T polymorphisms, and the haplotype with *ABCB1* 2677G > T/A, and 3435 C > T did not affect the edoxaban pharmacokinetics. This is consistent with previous pharmacogenomic studies of edoxaban indicating the lack of effect of *ABCB1* genotypes on the plasma trough concentration of edoxaban [17, 18]. Therefore, at least three *ABCB1* genotypes are unlikely to be associated with the efflux of edoxaban via P-gp.

The CYP3A5*3 polymorphism also had no effect on the edoxaban pharmacokinetics, which is consistent with a previous pharmacogenomic study of edoxaban [18]. These results support the finding that edoxaban is metabolized via CYP3A4/5 to form M-4, although the exposure of M-4 is < 10% of the total exposure of edoxaban [11].

The concomitant use of P-gp and/or CYP3A4 inhibitors (amiodarone, diltiazem, and verapamil) did not affect the CL/F of edoxaban, which is contrary to the results in previous drug-drug interaction studies [30, 31]. It was reported that concomitant use of quinidine, amiodarone, verapamil, and dronedarone increased the area under the concentration-time curve of edoxaban and its maximum plasma concentration, while concomitant use of these drugs did not increase its plasma concentration at 24 h after oral administration [30]. A previous PPK study indicated that the concomitant use of P-gp inhibitors (including quinidine, verapamil, dronedarone, ketoconazole, and erythromycin) had impacts on both the CL/F and bioavailability of edoxaban [22]. These results suggested that the impacts of P-gp and/or CYP3A4 inhibitors on both the CL/F and bioavailability of edoxaban may be associated with drug–drug interactions in intestinal absorption or metabolism via CYP3A4 in the small intestine and liver. In the present study, the effect of P-gp and/or CYP3A4 inhibitors on the bioavailability and CL/F of edoxaban could not be detected in our PPK model, as there were no data on the absorption phase.

Our study has several limitations. First, in the PPK analysis, the parameters such as Vd/F and absorption rate constant were not estimated due to only one sampling data on the elimination phase in each patient. Second, the population mean of CL_NR_/F could be underestimated due to a lack of data on urine and metabolite samples as well as one sampling data in each patient, which may lead to the potential biases in estimating its CL/F. However, considering the mental and physical burden of patients, it is difficult to obtain multiple sampling on the absorption phase and urine samples from patients in clinical practice. Third, the number of patients with CYP3A5*1/*1 genotype and patients treated with CYP3A4 and/or P-glycoprotein inhibitor were small. Therefore, the PPK and pharmacogenomic study of edoxaban in a larger number of Japanese population and plasma samples including metabolites in each patient should be conducted in the future to estimate parameters accurately and examine the effect of CYP3A5*3 genotype and CYP3A4 and/or P-glycoprotein inhibitor on edoxaban pharmacokinetics in more detail.

## Conclusions

Our results suggest that genetic polymorphisms of *CYP3A5* and *ABCB1* are not considered intrinsic factors for edoxaban pharmacokinetics in Japanese AF patients. These results may help to prevent adverse events without reference to genetic polymorphisms of *CYP3A5* and *ABCB1* and to provide individualized anticoagulant therapy with edoxaban.

## Data Availability

No datasets were generated or analysed during the current study.

## Abbreviations

AF: Atrial fibrillation
ALT: Alanine aminotransferase
AST: Aspartate aminotransferase
BW: Body weight
CLcr: Creatinine clearance
CL/F: Apparent oral clearance
CV: Coefficient of variation
CWRES: Conditional weighted residuals
CYP: Cytochrome P450
FXa: Activated coagulation factor X
IPRED: Individual predicted value
M-4: An edoxaban metabolite
NONMEM: Non-linear mixed-effects modeling
OBJ: Objective function value
OBS: Observed value
pcVPC: Prediction-corrected visual predictive check
P-gp: (ABCB1) P-glycoprotein
PPK: Population pharmacokinetic
PRED: Population predicted value
Scr: Serum creatinine
Vd/F: Apparent volume of distribution
